# MIR@NT@N: a framework integrating transcription factors, microRNAs and their targets to identify sub-network motifs in a meta-regulation network model

**DOI:** 10.1186/1471-2105-12-67

**Published:** 2011-03-04

**Authors:** Antony Le Béchec, Elodie Portales-Casamar, Guillaume Vetter, Michèle Moes, Pierre-Joachim Zindy, Anne Saumet, David Arenillas, Charles Theillet, Wyeth W Wasserman, Charles-Henri Lecellier, Evelyne Friederich

**Affiliations:** 1 Cytoskeleton and Cell Plasticity lab, Life Sciences Research Unit-FSCT, University of Luxembourg, L-1511 Luxembourg, Luxembourg; 2 Centre for Molecular Medicine and Therapeutics, Child and Family Research Institute, University of British Columbia, 950 West 28th Avenue, Vancouver, BC V5Z 4H4, Canada; 3 Structure and Function of the Cell Nucleus, Institute for Research in Immunology and Cancer (IRIC), Université de Montréal, Montréal (Québec), Canada; 4 Institut de Recherche en Cancérologie de Montpellier INSERM U896, Université Montpellier1, CRLC Val d'Aurelle Paul Lamarque, Montpellier, F-34298, France; 5 Institut de Génétique Moléculaire de Montpellier UMR 5535 CNRS, 1919 route de Mende, F-34293 Montpellier cedex 5, France

## Abstract

**Background:**

To understand biological processes and diseases, it is crucial to unravel the concerted interplay of transcription factors (TFs), microRNAs (miRNAs) and their targets within regulatory networks and fundamental sub-networks. An integrative computational resource generating a comprehensive view of these regulatory molecular interactions at a genome-wide scale would be of great interest to biologists, but is not available to date.

**Results:**

To identify and analyze molecular interaction networks, we developed MIR@NT@N, an integrative approach based on a meta-regulation network model and a large-scale database. MIR@NT@N uses a graph-based approach to predict novel molecular actors across multiple regulatory processes (i.e. TFs acting on protein-coding or miRNA genes, or miRNAs acting on messenger RNAs). Exploiting these predictions, the user can generate networks and further analyze them to identify sub-networks, including motifs such as feedback and feedforward loops (FBL and FFL). In addition, networks can be built from lists of molecular actors with an *a priori *role in a given biological process to predict novel and unanticipated interactions. Analyses can be contextualized and filtered by integrating additional information such as microarray expression data. All results, including generated graphs, can be visualized, saved and exported into various formats. MIR@NT@N performances have been evaluated using published data and then applied to the regulatory program underlying epithelium to mesenchyme transition (EMT), an evolutionary-conserved process which is implicated in embryonic development and disease.

**Conclusions:**

MIR@NT@N is an effective computational approach to identify novel molecular regulations and to predict gene regulatory networks and sub-networks including conserved motifs within a given biological context. Taking advantage of the M@IA environment, MIR@NT@N is a user-friendly web resource freely available at http://mironton.uni.lu which will be updated on a regular basis.

## Background

The cells of an organism harbor a common set of genes which are differentially regulated in time and space by various factors allowing them to adopt distinct phenotypes and to exert various functions. Among the regulators, transcription factors (TFs) and microRNAs (miRNAs) which are small 21-23-nucleotide-long, non-coding RNAs, play a cardinal role in the determination of cell fate and homeostasis, in physiological and disease conditions. While TFs act at the DNA level by binding to *cis*-regulatory elements of genes, termed Transcription Factor Binding Sites (TFBSs) [1-3], miRNAs regulate gene expression at the post-transcriptional level by binding to the 3'-untranslated region (3'-UTR) of messenger RNAs [4]. They thereby inhibit protein synthesis by triggering the degradation of the target messenger or by inhibiting its translation, contributing to the fine-tuning of gene expression [5,6]. Rather than acting independently or in parallel, it is now well established that TFs and miRNAs act in concert in networks to regulate target genes in a coordinated manner [7,8]. TFs and miRNAs are in turn regulated, in part, at transcriptional and post-transcriptional levels. In line, regulatory nodes may comprise TFs and miRNAs that form sub-networks including fundamental, evolutionary conserved regulatory motifs such as feedback or feedforward loops (FBL, FFL) [8-12], contributing to the modulation of gene expression and the adaptation of cells to changes in their environment. For example, these regulatory schemes play an important role in cell fate determination during embryonic development and during the differentiation/dedifferentiation processes of cells, conferring them genetic plasticity [13-15].

Potentially, a TF binds to the regulatory motifs of thousands of genes while a miRNA may target several hundreds of messenger RNAs. Consequently, *in silico *predictions of binding sequences of these regulators require additional filtering to identify those with potential biological relevance. In line, recent studies have demonstrated that combining binding site predictions with context-linked, experimental genome-wide co-expression data, is a powerful approach to identify biologically meaningful molecular interactions [7,12,16,17].

To date, databases and tools have been established which compile and explore experimentally supported and predictive data from TF regulations on coding genes (TF→Gene) [3,18,19], TF regulations on miRNA genes (TF→miRNA) [20-23] and miRNA regulations on messenger RNAs (miRNA→ gene), [21,24,25]. While these resources and associated tools are useful to predict TF or miRNA binding sites and associated molecular interactions, an approach which integrates this information at a genome-scale level to identify miRNA, TF and target gene regulatory sub-networks is still not available. Thus, a resource dedicated to the reconstitution of meta-regulation networks guided by '-omics' data would be of great interest to users to better understand how these regulations contribute to biological processes in normal and pathological conditions.

**Figure 1:**
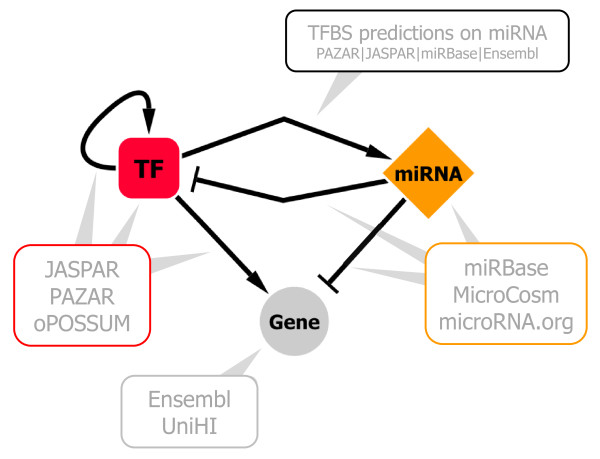
**Meta-regulation network model**. The MIR@NT@N meta-regulation network model illustrates interactions between three biological entities, transcription factors (TF), non coding microRNA genes (miRNA) and coding-genes (Gene). This Gene entity represents a target at multiple levels: a DNA sequence (TF regulations), a messenger RNA (miRNA regulations), or a protein (protein-protein interactions). Similarly, edges describe TF regulations (arrows) at DNA level, and miRNA regulations (blunt arrows) at RNA level. Squares represent TFs, diamonds miRNAs and circles coding-gene targets. The MIR@NT@N database is a large-scale resource which integrates information from multiple available databases: PAZAR, JASPAR and oPOSSUM (for TF regulations), miRBase, MicroCosm and microRNA.org (for miRNA target predictions), UniHI (for protein-protein interactions) and Ensembl (for gene annotations). Based on these resources, the MIR@NT@N database also integrates large-scale information about TF regulations on miRNAs through the prediction of TFBS on upstream sequences of miRNA precursors.

Here, we have developed MIR@NT@N (MIRna @Nd Transcription factor @nalysis Network), based on a graph-theoretical method to integrate multiple regulation levels into a unified model (Figure 1). MIR@NT@N predicts novel molecular actors and the form of their interplay. Based on these predictions or on lists of known molecular actors, users can generate regulatory networks and extract FBL and FFL sub-networks. Analyses can be contextualized and filtered by associating, for example, large-scale co-expression data. Collectively, MIR@NT@N offers novel applications to gain insight into the potential mechanisms of action of molecular regulators and their targets, in a given biological context.

## Implementation

### The MIR@NT@N application

The MIR@NT@N application is an open-access web interface, which can be accessed as a standalone module or through the workflow of M@IA, an environment dedicated to integrative biology analyses [26]. MIR@NT@N is built in the PHP programming language for database generation, data integration, analysis scripts (including graph construction and FBL and FFL detection) and interface. It also uses applications included in M@IA: R language (http://www.r-project.org) for statistical computing and Graphviz tool (http://www.graphviz.org) for interaction graph generation. Data can be further processed using any other module of M@IA, such as automatic gene annotation and data mining based on ontology or metabolic/signaling pathways. The MIR@NT@N application is connected to a MySQL relational database integrating information on biological entities and their regulations and interactions. The MIR@NT@N help main page includes an overall description of each section, including a quick tutorial and example files. To guide users in their analysis, MIR@NT@N also provides a contextual help available within each section, by explaining parameters, checking loaded data, and suggesting analysis refinement.

## Results and Discussion

### The MIR@NT@N database

Biological entities were identified and annotated in the MIR@NT@N database using three source databases: PAZAR (Release 2010, January 2010), which includes JASPAR (Release 2009, October 2009) [18,27,28], for TF annotations, including TFBS profiles as Position Frequency Matrices (PFMs); miRBase (Release 14, April 2010) [25] for the miRNA annotations including localization on genome sequence; and Ensembl (Release 56, October 2009) [29] for TF and coding-gene identifier mapping.

To integrate TF regulations on coding-genes (TF→Gene), we combined PAZAR [28] which provides public TF regulatory data, and oPOSSUM (Release 2.0, January 2007) [19], a large scale database which among other features, predicts TFBSs conserved between species, using TFBS profiles from the JASPAR database. Further, we extracted from oPOSSUM all TF→Gene regulations predicted in the 10 kb upstream and 5 kb downstream region of genes, with a score threshold of 0.85, and a high conservation level (top percentile of 0.010 and minimum identity of 80%). For each of the JASPAR profiles, we calculated the correspondence of the scores with empirically derived p-values for a common reference DNA sequence (see "Motif Scoring Procedure and Computation of JASPAR Profile Matrix Score p-values" section on MIR@NT@N website for more details) and established that, for 97% (127 of 130) of the binding site profiles, the applied 0.85 threshold corresponds to a p-value no more permissive than p < 0.01. Present databases [21-23] do not provide sufficient information about TFBSs within genes encoding miRNAs (TF→miRNA) required for building a large-scale meta-regulation model. TransmiR provides a limited number of experimentally validated regulations for multiple species [20,22]. MiRGen offers the downloading of large-scale predicted regulations, but only for Human and Mouse, and without TFBS scores and locations [21], whereas PuTmiR provides scores only for Human [23]. Regulation of transcription of coding and miRNA genes has been proposed to be similar. This is based on the observation that promoter regions of both share common features such as the presence of CpG islands and specific histone modification markers [30]. In further support of common regulatory mechanisms, it has been shown that a same transcription factor can regulate both, protein-encoding and miRNA genes [31]. Thus, we have used a standard TFBS detection algorithm [3] and TFBS profiles from the JASPAR database to predict TF→miRNA regulations on a large scale. PFMs were converted into Position Weight Matrices (PWMs) and used to predict potential TFBSs in 10 kb sequences located upstream of miRNA precursors, extracted from Ensembl database, according to pre-miRNA localization provided by miRBase. To limit the noise of false predictions, only predicted TFBSs with a score higher than 0.65 were integrated into MIR@NT@N database.

To refine the TFBS prediction on the miRNA upstream sequences, we provide additional information on TFBS location within "CpG islands" (CGI), regions which are frequently associated with promoter regions [30,32]. CGI were predicted (for Human, Mouse and Rat) with CpGcluster [33], a distance-based CGI-finder algorithm, and CpGProd [34], a tool that identifies promoter regions associated with CGI.

To integrate miRNA-dependent regulations (miRNA→gene) into MIR@NT@N database, we combined the miRBase Targets database, rebranded as MicroCosm (Release 5, September 2009) and hosted at the EBI (release 5), and microRNA.org (Release September 2008) [35]. Each resource can be used, through the MIR@NT@N application, separately with scores (from the minimum score of 13 to maximum score of 23 for MicroCosm, and from the minimum score of 140 to the maximum score of 205 for microRNA.org) derived from the miRanda algorithm (John et al., 2004), or simultaneously with a unified score (derived by a non-linear transformation and distributed uniformly between 0 and 1).

In addition, we integrated protein-protein interactions from the UniHI database [36], motivated by the idea that clustered miRNAs can coordinately regulate protein-protein interaction networks [37].

Thereby, for 7 species (*Caenorhabditis elegans*, *Danio rerio*, *Drosophila melanogaster*, *Gallus gallus*, *Homo sapiens*, *Mus musculus *and *Rattus norvegicus*), MIR@NT@N database contains 3 638 miRNAs, 335 TFs, 68 202 coding-genes as well as a large number of predicted interactions for a common standard score threshold of 0.85 (211 783 miRNA→Gene, 32 224 TF→miRNA and 273 264 TF→Gene).

The MIR@NT@N database is publically available on the website, which proposes 1) a dump file of the database in a SQL format, 2) a file (tab-delimiter format) with all TFBS scores calculated from miRNA upstream sequences and TF profiles from PAZAR, and 3) a file (tab-delimiter format) of the meta-regulation network, combining all regulations (TF→miRNA, miRNA→Gene and TF→Gene) for a common standard score threshold of 0.85.

### Overview on MIR@NT@N

The MIR@NT@N application works within a meta-regulation network model (Figure 1) in order to a) identify novel major regulators and targets based on an input list of actors, through interaction graph analysis and sub-network detection; and b) construct networks with well-defined actors with a presumed role in a given context.

Thus, two types of queries are involved. The first type allows searching for novel key actors in a biological context, using TF/gene/miRNA lists as input (including quantitative expression profiles generated by transcriptomics/proteomics experiments). This query includes three sections: (i) "Transcription Factor regulation" which statistically predicts potential TFs regulating a list of miRNAs, or conversely miRNAs regulated by a list of TFs; (ii) "miRNA regulation" which statistically predicts the significant targets of a list of miRNAs or the miRNAs targeting a list of genes; and (iii) "Regulation Network" which combines both TF and miRNA regulation predictions to reconstitute meta-regulation networks and allows detection of regulatory motifs such as FBL or FFL. The second type of query provides an overview on any TF, gene or miRNA, including their interactions: The "Quick Search" rapidly retrieves information on any actor, its regulators and/or targets, while the "Quick Network" generates regulation networks from a list of actors presumed to be involved in a particular biological context, and also allows the extraction of sub-networks including regulatory motifs.

As described below, the performance of MIR@NT@N was evaluated with published, experimentally validated data and further highlighted in a biological case study on epithelium to mesenchyme transition (EMT). EMT is an evolutionary conserved biological process involving the reprogramming of regulatory networks, including TFs, miRNAs and their targets, in epithelial cells during gastrulation, neural crest cell migration in embryogenesis. In adults EMT is reactivated in pathological situations such as wound healing, carcinoma progression, and fibrosis [14,38,39].

### Transcription Factor regulation

**Figure 2:**
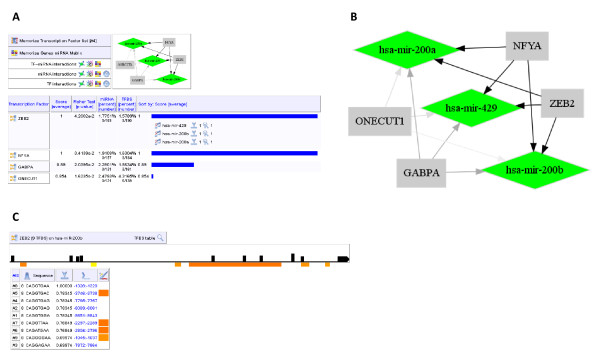
**Output of "Transcription Factor regulation"**. (A) Result of the query predicting TFs which potentially regulate the miR-200 family (cluster of down-regulated hsa-mir-200a, hsa-mir-200b, hsa-mir-429 represented here as down-regulated, green color code) with stringent criteria: quality score ≥ 0.85, number of miRNA by TF ≥ 3, and Fisher test p-value ≤ 0.05. The four predicted TFs are shown. Results are sorted by the quality score (lower panel). Information on all TF scores, including targeted miRNAs and the number of potential TFBS in a frame are given (shown only for ZEB2). All results, including generated graphs, can be visualized, memorized and exported in various formats. (B) Generated regulation graph with all input miRNAs shown here as down-regulated (diamonds in green) and predicted TFs (squares in gray). Edges represent regulations, and the gray canonical color code corresponds to the quality score. (C) Detail result interface showing the hsa-mir-200b upstream sequence (black line) with all TFBS (black boxes) predicted for ZEB2 and predicted promoter sequences (yellow or orange bar below the black line). A table ranks TFBS by quality score, and includes: "ID" (corresponding to the position in the sequence), sequence size, binding sequence, quality score, localization (within the upstream sequence and the genome through a hypertext link), and potential localization within a predicted promoter (orange boxes).

This section reports potential TF→miRNA regulation given a list of TFs or miRNAs to identify novel TF regulators and miRNA targets. The result is a table of TFs or miRNAs, filtered and ranked by their relevance according to several criteria (Figure 2A) including: the quality score, the Fisher test p-value, the number of TFBSs and TF→miRNA regulations. Results can be visualized through an interaction graph (Figure 2B) using a gray scale canonical color code to convey prediction scores. To facilitate the detection of regulatory clusters, the graph includes expression information (if provided as input) using the green/red canonical color code. In addition, to identify clusters of miRNAs regulated by the same TFs, or clusters of TFs which regulate the same miRNAs, an analysis of the interaction graph provides "square" and "curvature" graphs [26]. To refine the prediction analysis, all corresponding TFBSs can be visualized through a user-friendly interface (Figure 2C) which provides the binding sequence, its length, the quality score, the localization on the miRNA upstream sequence and in the genome (with a link to Ensembl), and information about predicted promoters using a canonical color code for prediction scores (from yellow to red). All results (tables and graphs) provide links to external knowledge sources (PAZAR for TFs, miRBase for miRNAs, Ensembl for genes and TFBS localization). Results can be exported and stored for further analysis, using for instance the M@IA environment [26] or external applications.

To illustrate the performances of these functions, we identified TFs predicted to regulate the miR-200 family, including miR-200a, miR-200b and miR-429, which are important for the maintenance of the epithelial phenotype and in the prevention of EMT [40]. Using stringent criteria we identified four TFs (Figure 2AB) including ZEB2 which has recently been reported to directly interact with E-boxes of the miR-200 promoter [14]. The predicted TFBSs of ZEB2 can be located on the miR-200 promoter by clicking on the ZEB2 table, yielding 1 to 9 sites with the criteria 0.9 and 0.65, respectively (Figure 2C). Interestingly, one of the predicted TFBSs is located within the experimentally identified region of the miR-200 promoter (Bracken et al., 2008) shown to be negatively regulated by the related transcription factor ZEB1, mediated through paired E-boxes.

### miRNA regulation

**Figure 3:**
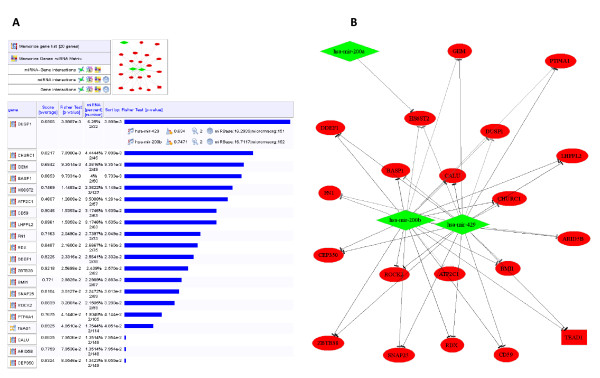
**Output of "miRNA regulation"**. (A) Result of the prediction of target genes for the miRNA 200 family (cluster of down-regulated hsa-mir-200a, hsa-mir-200b, hsa-mir-429) with medium stringency criteria (miRBase score ≥ 16 and p-value ≤ 0.05, microRNA.org score ≥ 150, at least two miRNAs per gene). The output list was filtered with a list of 132 up-regulated genes. The 20 predicted target genes presented in the table (lower panel) were sorted by the Fisher test p-value. Information can be obtained for each target gene, including its miRNA regulators and unified prediction scores (panels for the first gene is shown). (B) Generated regulation graph with all input miRNAs shown as down-regulated (diamonds in green) and predicted target genes shown as up-regulated (ellipses and square in red). Edges represent regulations, and the gray canonical color code corresponds to the quality score.

This section determines potential miRNA→Gene regulations from a list of miRNAs or other genes to identify novel actors, i.e. miRNA regulators and targeted genes. The result is a table of genes filtered and ranked by their relevance to the input list of miRNA, using alternative criteria (Figure 3A): MicroCosm and/or microRNA.org scores (or corresponding unified score), Fisher test p-values, number of targets per miRNA and the number of targeted sequences (boxes) per gene. Inversely, this section can provide, using the same parameters, a list of miRNAs predicted to regulate a given list of genes.

As described above in the "TF regulation" section, results can also be filtered using a specific list of data to contextualize the study, visualized through the same type of interaction graphs (Figure 3B). We illustrated this feature by predicting genes that are potentially targeted by three miR-200 family members. MIR@NT@N predicted 934 genes to be at least targeted by two miR-200 family members, using the criteria described in legend of Figure 3. As these miRNAs are known to be down-regulated in EMT [40], we contextualized the study with a biological filter using a list of 132 genes found to be up-regulated in experimentally induced EMT [17,41], reasoning that messengers with negatively correlated expression levels may be targets of the miR-200 family [17,41]. Twenty genes were predicted to be targeted by miR-200 family members (Figure 3A and 3B). The list included FN1, an experimentally validated target of miR-200 [42], genes reported to play an important role in EMT [43,44] as well as genes with so far no described role in this process, yielding valuable hypotheses for experimental investigations.

### Regulation network generation

**Figure 4:**
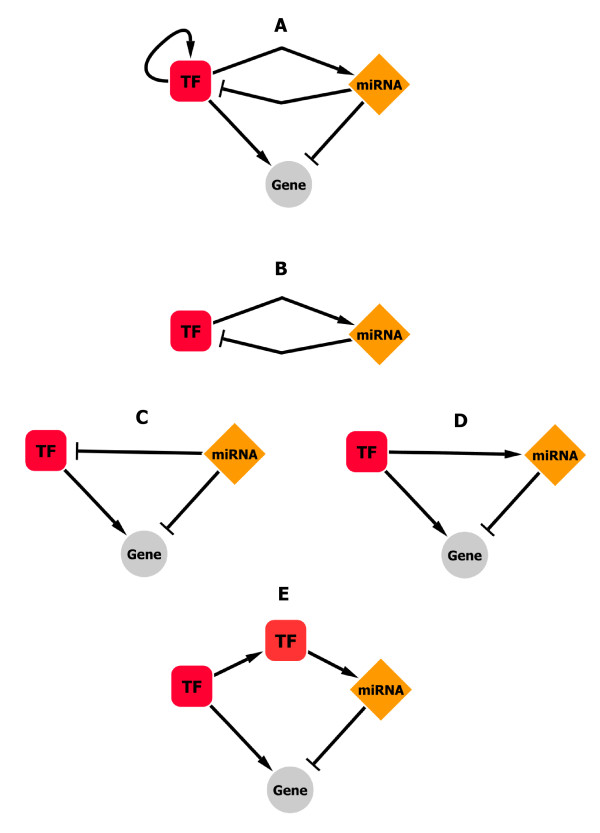
**Meta-regulation Network motifs**. (A) The meta-regulation network model integrates TFs, miRNAs and target genes with their regulations. This model allows describing two biologically relevant systems (FBL and FFL) that can be modelled as "network motifs". (B) The FBL "network motif" model describes a reciprocal regulation between a TF and a miRNA. (C, D, E) The FFL "network motif" model that includes additional target genes, can be illustrated as three distinct models: the FFL-miRNA model describes the regulation of a TF and a targeted gene by the same miRNA (C), the FFL-TF model describes the regulation of a miRNA and a targeted gene by the same the TF (D) and the FFL-indirect model integrates an additional TF in the FFL-TF (E).

This section combines "TF regulation" and "miRNA regulation" interfaces to allow the construction of meta-regulation networks (Figure 4A), with an orientation towards the detection of network motifs and the identification of multiple target genes, for both TFs and miRNAs. Within a specific context, the user may identify, from a list of miRNAs, both novel molecular actors and the nature of the regulation, highlighting fundamental regulatory motifs [10]. These motifs include FBLs consisting in a reciprocal regulation of a TF and a miRNA (Figure 4B), the TF controlling the miRNA and the miRNA regulating the TF [45]. The FBL modulates the activity of regulators, which is crucial for the spatio-temporal control of their function. On the other hand, a FFL is a regulatory system in which a regulator A regulates another regulator B, and both regulators regulate a common target C [10,11,46]. In MIR@NT@N, FFLs can involve a miRNA regulator (FFL-miRNA, Figure 4C) or a TF regulator (FFL-TF, Figure 4D). In addition, MIR@NT@N includes the concept of indirect FFLs (Figure 4E) in which the regulation of the miRNA by the TF is exerted by an intermediate TF.

Users can inform the system by providing a list of molecular interactions. For example, the user can use a list of miRNA→gene interactions experimentally inferred from microarray data combining genes and miRNA expression or a list of published TF→miRNA interactions. For this purpose, published and experimentally validated TF→miRNA interactions [20,22,23] are provided and can be used as a filter.

**Figure 5:**
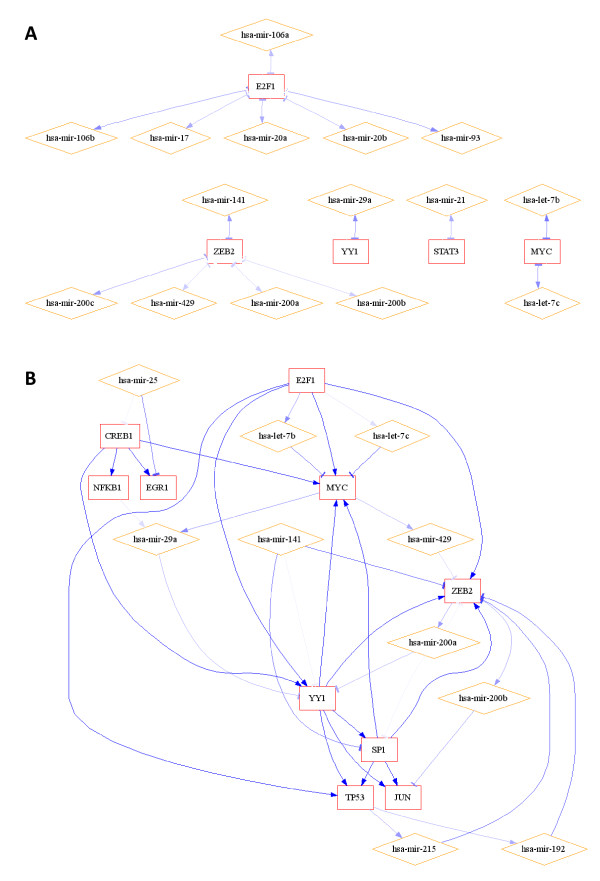
**Validation of FBL and FFL motif predictions**. 108 published TF→miRNA regulations (Human) were identified from Qiu and colleagues [22] for the 19 TFs in common with MIR@NT@N database. FBL (A) and FFL motifs B) were identified from the input list of miRNAs included in Qiu database, and by filtering actors with the 19 common TFs and with the 108 published TF→miRNA regulations. Non stringent criteria were used for TF regulation predictions (TFBS score ≥ 0.65, TFBS length ≥ 6, number of miRNA per TF ≥ 1, genes targeted by at least 1 TF), and to filter miRNA regulation predictions we used criteria corresponding to a unified score ≥ 0.8 (miRBase score ≥ 17 and p-value ≤ 0.01, and MicroRNA.org score ≥ 152), and 1 gene per miRNA. Squares represent TFs, diamonds represent miRNAs, and edges represent predicted regulations (blue scale color code used for the prediction score). For FBL sub-network, edges represent double regulations.

To demonstrate regulatory motif detection, we analyzed TF→miRNA regulations from published data by Qui et al., including TransmiR data [20,22]. For the 19 human TFs found in common within the MIR@NT@N and Qiu databases, we observed that 81% of the interactions listed in the Qiu database were predicted by MIR@NT@N with a TFBS score higher than 0.65, and 43% with a TFBS score higher than 0.85 (Additional file 1). Using entire MIR@NT@N database, we extracted putative FBLs (Figure 5A) and FFLs (Figure 5B), including well-documented FBLs implicating an E2F TF family member and several miRNA families [47,48], the ZEB "Zinc Finger E-Box" TF family and the miR-200 family [49,50], and YY1 and hsa-mir-29a [51]. Moreover, we predicted hsa-mir-29a to be regulated by NFKB1 and MYC (Figure 5B), consistent with previous reports [51,52] and only recently identified to be co-regulators of their common target mir-29a [53].

Collectively, these results underline the efficiency of MIR@NT@N to generate an overview of a regulatory network and to detect core sub-networks within a biological system.

### Quick Search and Quick Network interfaces

Quick search and Quick network interfaces allow searching for regulations between known or assumed actors of a biological context. The "Quick Search" section is a full text search engine that provides data pertinent to specific entities (miRNA, TF or target gene). Information about each biological item is available through hypertext links to external data sources (Ensembl for genes, PAZAR for TFs and miRBase for miRNAs). Potential TF/miRNA regulations and predicted TFBSs are accessible through an internal MIR@NT@N application pipeline.

**Figure 6:**
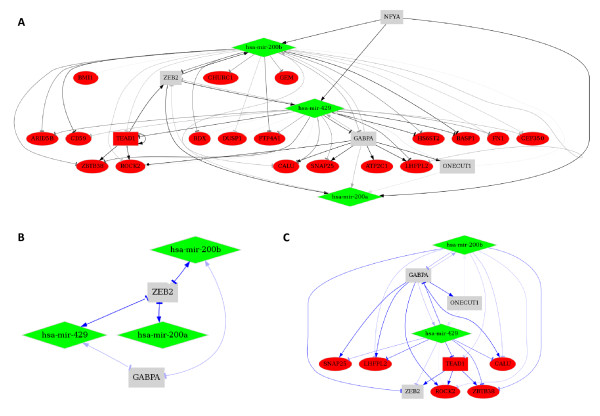
**Regulatory networks in EMT generated by the Quick Network interface**. (A) Networks were generated using the down-regulated miR-200 family (diamonds in green), the four TFs (squares in gray) predicted to regulate these miRNAs and the twenty predicted up-regulated target gene list (ellipses in red) predicted to be regulated by these miRNAs. Scores of 0.85 or 0.8 were used for TF regulations or miRNA regulations, respectively. (B) FBL extracted from the previous network (A), showing a regulation loop between ZEB2 and miR-200 family. (C) FFL detected from the previous network (A), focusing on genes targeted by both, TFs and miRNAs.

The "Quick network" is a powerful application to extract information from a list of TFs, miRNAs and other genes with a presumed function within a biological context, as supported by literature or experimental data. The user can retrieve corresponding regulatory predictions and generate a network of predicted interactions as a comprehensive graph, yielding information on the interaction mechanisms of the analyzed actors. Functional motifs (FFL and FBL) can be detected to identify major actors and targets organized into regulatory sub-networks. The respective quality score thresholds of TF and miRNA regulations can be modulated through a cursor and information about protein interactions can be integrated into the network (described as experimentally validated in UniHI). A cross-species network analysis is possible by selecting different species associated with the input symbol (e.g. the input symbol "hsa-mir-200a" will be changed into "mmu-mir-200a" if the "Mus musculus" species is selected). The output is an exportable interaction graph recapitulating all predicted interactions and which is linked to external resources (Figure 6).

The miR-200 family served as an example to illustrate how the "Quick Network" interface generates regulatory networks in a given context (Figure 6). The generated network recapitulates the results described above and integrates the predictions of the TFBS on coding genes (Figure 6A). The FBL function suggests the presence of a double negative FBL between mir-200a, mir-200b, mir-429 and ZEB2 (Figure 6B), as is described in the literature [54]. Target genes already described in EMT, such as ROCK2 [44] and TEAD1 [55] were highlighted from the FFL network (Figure 6C).

### Future extensions of MIR@NT@N

MIR@NT@N, which takes advantage of the M@IA environment [26], can be readily extended to include additional miRNA target prediction databases (such as TargetScan [56] or PicTar [57]) or more TF binding profiles from collections that use a standard PFM format. The PWM methods utilized within MIR@NT@N are well-established, but likely to be replaced with more advanced models in the near future. High-throughput sequencing coupled to chromatin immunoprecipitation now routinely generates collections of ~103 binding sites, providing richer descriptions of binding properties of TFs. New algorithms are emerging which build on such data to describe patterns using higher-order models to account for interactive effects between positions. However, the rapidly emerging changes have not stabilized, so we applied the established methodology within the source database in the oPOSSUM system. We intend to upgrade MIR@NT@N when a new motif scoring procedure is supported by the JASPAR database of binding profiles.

Moving forward, novel data classes will be implemented into MIR@NT@N, such as histone modifications or alternative splicing that play central roles in gene expression and for which databases are already available [58,59]. We will incorporate more knowledge sources, such as known promoter sequences and experimentally validated TF-miRNA regulations [20,22,60].

## Conclusions

Here, we described MIR@NT@N, available as an open-access web application at http://mironton.uni.lu, which identifies meta-regulation networks implicating TFs, miRNAs and target genes. The possibility to predict TF- and miRNA-mediated regulations at a genome-wide scale is an important novel feature of MIR@NT@N. MIR@NT@N facilitates the analyses of "-omics" data (i.e. any experiment made at a genome scale such as transcriptomics and proteomics analyses) and allows detection of relevant molecular interactions and associated regulatory motifs (e.g. FFL). Users analyzing complex spatio-temporal gene regulation data can obtain experiment-suitable insights into the regulatory mechanisms governing cellular processes.

## Availability and requirements

**Project name**: MIR@NT@N

**Project home page**: http://mironton.uni.lu

**Operating system(s)**: Platform independent

**Programming language**: PHP, HTML, Javascript, R

**Other requirements**: M@IA environment including Apache 1.3 or higher, MySQL 4.0 or higher, R 2.0 or higher, Graphviz

**License**: GNU GPL

**Any restrictions to use by non-academics**: licence needed

## List of Abbreviations

TF: Transcription Factor; PFM: Position Frequency Matrices; PWM: Position Weight Matrices; TFBS: Transcription Factor Binding Site; miRNA: microRNA; CGI: CpG Island; FBL: Feedback loop; FFL: Feedforward loop; EMT: Epithelium to Mesenchyme Transition;

## Authors' contributions

ALB designed the MIR@NT@N approach and the software. EP-C and WWW helped in JASPAR/PAZAR incorporation and TFBS predictions, contributed constructive suggestions to the study and to the manuscript. C-HL, GV, and MM contributed knowledge on the molecular biology of TF/miRNA regulations and EMT. DA and WWW contributed to the computation of JASPAR profile matrix score p-values. AS, EF, P-JZ and CT provided intellectual support and discussed the results. ALB, EF and C-HL wrote the manuscript. All authors read and approved the final manuscript.

## Supplementary Material

Additional file 1 **MIR@NT@N predictions for TF→miRNA regulations in Qiu et al**. Table providing MIR@NT@N database predictions (maximum score, maximum length and number of TFBS) for TF→miRNA regulations described in Qiu et al., for the 19 human TFs found in common. Click here for file
