# Supplementary material for: MIR@NT@N: a framework integrating transcription factors, microRNAs and their targets to identify sub-network motifs in a meta-regulation network model
Source: BMC Bioinformatics. 2011 Mar 4;12:67. doi: 10.1186/1471-2105-12-67 (PMC3061897; doi:10.1186/1471-2105-12-67)
Supplement: Additional file 1 — MIR@NT@N predictions for TF→miRNA regulations in Qiu et al. Table providing MIR@NT@N database predictions (maximum score, maximum length and number of TFBS) for TF→miRNA regulations described in Qiu et al., for the 19 human TFs found in common. [file 1471-2105-12-67-S1.PDF]

TF	miRNA	maximum score	maximum length	number of TFBS
CREB1	hsa-mir-132	1	9	19
E2F1	hsa-mir-106b	1	8	7
E2F1	hsa-mir-25	1	8	7
E2F1	hsa-mir-93	1	8	7
EGR1	hsa-mir-106a	1	8	39
MYC	hsa-mir-17	1	11	11
MYC	hsa-mir-18a	1	11	11
MYC	hsa-mir-19a	1	11	11
MYC	hsa-mir-19b	1	11	11
MYC	hsa-mir-20a	1	11	11
MYC	hsa-mir-92a	1	11	11
NFKB1	hsa-mir-125b	1	8	16
NFKB1	hsa-mir-146a	1	8	15
NFKB1	hsa-mir-155	1	11	12
NFKB1	hsa-mir-9	1	11	13
SP1	hsa-mir-106a	1	13	106
SPI1	hsa-mir-146a	1	12	32
SPI1	hsa-mir-23a	1	12	34
TP53	hsa-mir-125b	1	10	38
TP53	hsa-mir-34a	1	10	63
YY1	hsa-mir-29a	1	8	37
YY1	hsa-mir-29b	1	8	36
YY1	hsa-mir-29c	1	8	35
ZEB2	hsa-mir-200a	1	8	8
ZEB2	hsa-mir-200b	1	8	9
ZEB2	hsa-mir-429	1	8	8
TP53	hsa-mir-145	0.990481152	10	89
MYC	hsa-mir-106a	0.976419881	11	7
TP53	hsa-mir-34c	0.951661123	10	28
TP53	hsa-mir-34b	0.947878844	10	27
CEBPA	hsa-mir-223	0.94354	12	43
CEBPA	hsa-mir-1	0.92698	11	13
TP53	hsa-mir-155	0.926114513	10	15
TP53	hsa-mir-215	0.923259324	10	34
MYC	hsa-mir-23a	0.916826309	11	7
E2F1	hsa-let-7i	0.905419974	8	7
TP53	hsa-mir-192	0.900468461	10	56
JUN	hsa-mir-21	0.899708629	8	49
E2F1	hsa-mir-106a	0.889916263	8	8
E2F1	hsa-mir-18b	0.889720806	8	8
E2F1	hsa-let-7a	0.889613303	8	7
E2F1	hsa-mir-20b	0.88874782	8	8
E2F1	hsa-mir-363	0.888573108	8	8
MYC	hsa-mir-22	0.886947923	11	5
MYC	hsa-mir-29a	0.880321383	11	1
E2F1	hsa-let-7b	0.868385904	8	4
MYC	hsa-mir-429	0.84247595	11	5
HNF1A	hsa-mir-194	0.833360209	10	21
MYC	hsa-mir-195	0.830298874	11	5

NFKB1	hsa-mir-29a	0.817529184	13	16
NFKB1	hsa-mir-29b	0.815693881	13	17
MYC	hsa-mir-34a	0.802726251	11	3
MYC	hsa-let-7b	0.802412695	11	4
MYC	hsa-mir-23b	0.798065687	8	7
E2F1	hsa-mir-223	0.796464188	8	2
MYC	hsa-let-7g	0.796343766	8	2
MYC	hsa-let-7i	0.79564599	11	4
MYC	hsa-let-7a	0.789363985	11	6
ZEB2	hsa-mir-141	0.788878531	8	6
ZEB2	hsa-mir-200c	0.788327182	8	6
MYC	hsa-let-7f	0.788186126	11	6
MYC	hsa-let-7c	0.784454773	8	1
MYC	hsa-let-7d	0.779879856	11	3
ESR1	hsa-mir-222	0.769018158	13	1
ESR1	hsa-mir-221	0.767367714	13	1
MYC	hsa-let-7e	0.748582784	11	2
NFKB1	hsa-mir-29c	0.745005294	13	12
E2F1	hsa-mir-16	0.744532594	8	1
E2F1	hsa-mir-15a	0.744315169	8	1
E2F1	hsa-mir-19b	0.743182955	8	6
E2F1	hsa-mir-20a	0.742461169	8	6
E2F1	hsa-mir-92a	0.74245816	8	4
E2F1	hsa-mir-19a	0.739875318	8	6
E2F1	hsa-mir-18a	0.739125344	8	6
E2F1	hsa-mir-15b	0.738184297	8	5
E2F1	hsa-mir-17	0.737783442	8	6
STAT3	hsa-mir-21	0.727089683	12	1
MYC	hsa-mir-29c	0.727072612	8	4
RUNX1	hsa-mir-27a	0.725048904	8	1
MYC	hsa-mir-141	0.723534733	8	2
MYC	hsa-mir-221	0.723515239	11	2
NR1H4	hsa-mir-34a	0.717795811	10	2
E2F1	hsa-let-7c	0.712599888	8	3
MYC	hsa-mir-26a	0.71197061	11	3
E2F1	hsa-mir-195	0.708697212	8	8
E2F1	hsa-let-7d	0.677784493	8	2
MYC	hsa-mir-16	0.662113697	11	1
MYC	hsa-mir-15a	0.661401397	11	1
E2F1	hsa-mir-449	NA		
ESR1	hsa-mir-106a	NA		
ESR1	hsa-mir-17	NA		
ESR1	hsa-mir-18a	NA		
ESR1	hsa-mir-18b	NA		
ESR1	hsa-mir-19a	NA		
ESR1	hsa-mir-19b	NA		
ESR1	hsa-mir-20a	NA		
ESR1	hsa-mir-20b	NA		
ESR1	hsa-mir-21	NA		
ESR1	hsa-mir-363	NA		

ESR1	hsa-mir-92a	NA	
MYC	hsa-mir-200	NA	
REST	hsa-mir-21	NA	
TLX1	hsa-mir-17	NA	
TLX1	hsa-mir-18a	NA	
TLX1	hsa-mir-19a	NA	
TLX1	hsa-mir-19b	NA	
TLX1	hsa-mir-20a	NA	
TLX1	hsa-mir-92a	NA	
